# Intersectionality and Long Covid: Understanding the Lived Experiences of Ethnic Minority Groups in the United Kingdom

**DOI:** 10.1111/hex.70413

**Published:** 2025-09-04

**Authors:** Yojana Lotankar, Anna Cheshire, Damien Ridge, Carolyn Chew‐Graham, Nina Smyth, Claudia R. Knowles, Dipesh Gopal, Tom Kingstone, Shoba Dawson

**Affiliations:** ^1^ Psychology, School of Social Sciences University of Westminster London UK; ^2^ School of Medicine, Faculty of Medicine and Health Sciences Keele University Keele UK; ^3^ Health Psychology Section, Department of Psychology, Institute of Psychiatry, Psychology, and Neuroscience King's College London London UK; ^4^ Wolfson Institute of Population Health Queen Mary University of London London UK; ^5^ Research & Innovation Department Midland Partnership University NHS Foundation Trust Stafford UK; ^6^ Centre for Academic Primary Care, Bristol Medical School University of Bristol Bristol UK; ^7^ School of Medicine and Population Health University of Sheffield Sheffield UK

**Keywords:** Covid‐19, ethnic minorities, gender, intersectional, long Covid, qualitative methods, secondary analysis

## Abstract

**Introduction:**

Long Covid is the patient‐preferred term to describe persistent symptoms experienced following an acute Covid‐19 infection. The severity and unpredictable nature of long Covid symptoms can affect every aspect of an individual's life. Under‐represented groups such as ethnic minorities and lower socio‐economic groups are disproportionately affected by long Covid and often face challenges in accessing healthcare and additional support. This study employed an intracategorical intersectionality approach to explore how the diverse experiences of long Covid among people from ethnic minority backgrounds are influenced by complexities like gender and socio‐economic factors.

**Methods:**

A secondary analysis of 31 semi‐structured interviews with individuals with long Covid from ethnic minority backgrounds, using reflexive thematic analysis.

**Results:**

Findings are presented around the themes: (i) gender and ethnicity; (ii) socio‐economic factors and illness experience; and (iii) comorbidities, disabilities and living with long Covid. Participants describe challenges in gaining support, including from health professionals, family and communities, but these challenges were gendered to a degree, with women and men describing distinctive difficulties. Financial capacity was considered important in determining the type and extent of accessible care. Support from employers also influenced participant's ability to take adequate time to recover and return to work. The interplay of comorbidities with long Covid could heighten the risk of more severe symptoms and complicate help‐seeking for ‐and management of ‐long Covid.

**Conclusion:**

An intracategorical intersectional exploration of lived experience is necessary to reveal the nuances in individual experiences of long Covid. Findings will be of interest to health professionals and researchers in supporting their understanding of intersectional experiences of their patients.

**Patient Public Involvement:**

The HI‐COVE study was informed throughout by patient and expert advisory groups composed of individuals from ethnic minority backgrounds living with long Covid, their carers and professionals interested in this topic.

## 1 Introduction

On March 11 2020, the World Health Organization (WHO) declared the coronavirus disease (Covid‐19) a pandemic. This pandemic exacerbated pre‐existing health inequalities by disproportionately impacting the health and mortality rates of people from socio‐economically deprived backgrounds, those from ethnic minority communities [1], older people and people with disabilities [2].

After initial Covid‐19 infection, around 10%–20% of people reported experiencing prolonged symptoms, which became known as post‐Covid‐19 condition [3, 4] or long Covid—the patient‐defined term [5]. Data from the Office for National Statistics suggested that in April 2024 around 3.3% of the population living in private households in England and Scotland were experiencing long Covid symptoms [6, 7, 8]. According to PaRIS data, in 2024, approximately 7% of primary care patients aged 45 years and older reported ever having long Covid, while an estimated 5% reported living with persisting long Covid symptoms [9]. Long‐Covid symptoms are irregular and vary in severity, although common symptoms include extreme fatigue, chest pain, upper respiratory symptoms, muscle and joint pain, gastrointestinal symptoms, headache and neuro‐cognitive difficulties [7]. Symptoms often significantly alter people's ability to work, socialise and look after themselves [10], impacting mental health [11] and self‐identity [12]. Additionally, the lived experience of long Covid is shaped by varying social identities and life circumstances. For example, a good support system plays a crucial role by assisting in managing long Covid symptoms and gaining quality care [13]. However, fear of prejudice can prevent people from seeking help from their community due to anticipated stigma [14].

Ethnic minority groups are disproportionally affected by long Covid. Evidence points to a higher risk of developing long Covid among people of ethnic minority groups [15, 16]. However, one UK study found that people from ethnic groups had lower risks of developing long Covid symptoms [17]. Additionally, people from ethnic minority groups may have worse physical and mental health outcomes from long Covid [18]. They also have lower awareness of long Covid and relevant services, which may impact care‐seeking [19]. They are under‐represented in long Covid‐specialised services, such as UK ‘Long COVID clinics’, and are more likely to seek these services at later stages of symptom onset [20, 21]. Furthermore, individuals with long Covid from ethnic minority groups are known to encounter discrimination within healthcare settings and face significant challenges in navigating services, contributing to mistrust of the healthcare system and barriers to accessing care [14]. Medical racism is seen as a major public health concern [22], and its impact with respect to long Covid is reflected in such problems as reduced vaccine uptake rates (which lessen the risk of long Covid)—where those refusing the vaccine report more experiences of racial/ethnic discrimination in healthcare settings [23].

Other social groups are also at higher risk of developing long Covid and are disproportionately affected, including females, older people, those with pre‐existing comorbidities, gay/bisexual people and/or those living in deprived areas [24, 25, 26]. As Mullard et al. [27] demonstrated, people with long Covid from underprivileged communities are prone to getting ‘caught in a liminal space of misrecognition, invalidation and ambiguity’. Middle‐aged women (40–60 years old) are also susceptible to developing more debilitating long Covid symptoms and are less likely to recover quickly [28]. Moreover, long Covid symptoms may be triggered by, or cause, complications in the menstrual cycle, pregnancy and menopause [29]. The elevated risk for people with pre‐existing disabilities is possibly due to the elevated risk of exposure to Covid‐19 and barriers to receiving timely care during the infection [30]. Individuals residing in economically disadvantaged areas are more likely to develop long Covid, regardless of their initial risk of exposure to Covid‐19 infection [31]. Financial hardship and unstable employment conditions, frequently resulting from the diminished capacity to return to work due to long Covid symptoms, exacerbate the psychological distress experienced by individuals with the condition [32]. Finally, the unpredictable nature of long Covid symptoms has a distinct impact on people's lives and prospects, especially the young, for example, by impacting their participation in learning activities [33]. Management guidelines need to be developed using the full range of illness and lived experiences of those living with long Covid [4]. However, we know these groups and their experiences are already under‐represented in studies on Covid‐19 and long Covid [34, 35].

While some studies concentrate on the experiences of long Covid across specific dimensions, like ethnicity or gender, it is important to consider the impact of combinations of these wider dimensions on the lived experience of long Covid [36]. For example, Smyth et al. [14] found that ethnic minority women in particular believed that their symptoms were inclined to be invalidated as overreaction on their part and dismissed as excessive worry by health professionals. Intersectionality is a framework used to highlight the complex and distinct implications arising from overlapping identities and social positions (like ethnicity, gender and class) [37, 38], highlighting how such dimensions interact to create unique experiences of privilege, oppression and discrimination [39]. In health research, intersectionality has been used to reveal how multiple inequalities shape the experiences of living with an illness at individual and systemic levels [39, 40]. For example, lived experience, stigma [41, 42, 43] and discrimination linked to ethnic minority individuals' different social positions can hinder access to—and quality of—care [44].

The current study utilises the intracategorical intersectionality approach [41], which focuses on examining complexities and lived experiences within a specific social group. In the case of the current paper, this pertains to ethnic minorities living with long Covid, while acknowledging the simultaneous role of other social dimensions [42]. Intracategorical intersectionality shows that not everyone has the same experience in any defined group. In this paper, we examine differences within ethnic minority groups along various dimensions (e.g., gender, finances and ability), one at a time. The approach retains the importance of categorical boundaries (like ethnicity and gender), but explores nuances related to such dimensions critically: Revealing internal diversity and complexity [45]. While the intracategorical approach has been criticised as resembling descriptive narrative, we instead agree with researchers who point to the importance of understanding the complex real‐life interplays of marginalisation and dominant social identities/positions, and the possibilities of agency therein [42]. Importantly, this approach acknowledges how power dynamics involved in engaging with institutions depend on an individual's social identities and locations, helping to create distinct experiences of living with an illness [43, 46]. Such an intersectional approach not only highlights health disparities but also potentially provides a better explanatory framework for their existence and thus potential remedies [47].

There is a lack of research on the impact of intersectionality on the lived experience of long Covid [36]. Thus, in this paper, we apply an intersectional lens to a qualitative secondary data analysis [45] of transcripts of interviews conducted for a study of individuals from minority ethnic backgrounds who self‐identified as experiencing long Covid [14, 48, 49]. We examine how gender, class, ability, age and social circumstances intersect with ethnicity [50] to shape agency, as well as how institutions shape individuals' experiences of living with long Covid and their management of the condition.

## 2 Methods

### 2.1 Design

This paper reports on a secondary data analysis of interview data carried out for the HI‐COVE study [49]. Using semi‐structured interviews, the HI‐COVE study employed a decolonising lens to explore the lived experiences of people of ethnic minorities with long Covid, who are disproportionately affected by acute Covid‐19 infection, at higher risk for developing long Covid [15, 51], and have shared experiences of accessing healthcare. The study design and methodology were guided by a patient advisory group, which consisted of people (one male and six females) living with or caring for someone with long Covid who were from ethnic minority backgrounds. Additionally, an expert advisory group comprised of six researchers who were interested in long Covid and the health of ethnic minority populations [49]. The study was approved by the University of Westminster Ethics Committee (Ref: ETH2122‐1074) and included consent to re‐analyse the data.

### 2.2 Participants and Recruitment

People aged 18 years and over, living in the United Kingdom, from ethnic minority backgrounds, and who self‐reported having long Covid were recruited via advertisements on social media, university sites, and community networks and spaces. A screening checklist developed per criteria set by the WHO [46] was used to confirm that the potential participants were experiencing symptoms consistent with long Covid. Participant demographics are presented in Tables 1 and 2.

**Table 1:** Participant characteristics.

Characteristic	*N*	Percentage (%)
Gender		
Male	15	48.4
Female	16	51.6
Ethnicity background		
Arab	3	9.7
Black	10	32.2
South Asian	10	32.2
Mixed heritage	6	19.4
Other	2	6.5
Age range (years)		
20–29	9	29.0
30–39	10	32.2
40–49	6	19.4
50–59	4	12.9
> 60	1	3.2
Missing	1	3.2
Occupational background		
Student or not employed	7	22.6
Healthcare sector	4	12.9
Educational/professional sector	10	32.2
Transport sector	3	9.7
Sales/customer services	4	12.9
Skilled trade	2	6.5
Missing	1	3.2
Ladder for community standing*		
1–3	3	9.7
4–7	20	64.5
8–10	3	9.7
Did not want to answer	5	16.1

* Subjective social status measured using the MacArthur Scale of Subjective Social Status ladder (Goodman et al. 2001); a higher score represents higher social standing in a person's community.

**Table 2:** Year of first Covid‐19 infection.

Year	*N*	Percentage (%)
2020	14	45.2
2021	11	35.5
2022	5	16.1
Unknown	1	3.2

### 2.3 Data Collection

Between June 2022 and June 2023, 31 in‐depth one‐to‐one semi‐structured interviews were conducted by five researchers—three White females, one South Asian female and one White male. Reflexive positions of all interviewers and authors can be found in the supporting files (see Supporting file 1). An interview topic guide was modified iteratively as data were generated and analysed to facilitate the collection of overlooked narratives of living with long Covid. Interviews were audio recorded, transcribed verbatim and anonymised.

### 2.4 Data Analysis

Secondary analysis using reflexive thematic analysis [52] was completed in four phases: (1) After the lead author familiarised herself with the data, initial codes relating to intersectional experiences were identified inductively, reviewed by the co‐authors and finalised. (2) Transcripts were coded in NVivo [53] according to the draft coding framework. Each code was then reviewed and new codes were suggested, discussed and finalised with co‐authors; transcripts were re‐coded with the additional codes. (3) Next, data coded under socio‐demographic codes (age, gender, class, religion, comorbidity/disability and sexuality), particularly data that fell under dual socio‐demographic codes (e.g., ‘gender’ and ‘class’), were explored. Additionally, codes describing experiences such as ‘discrimination’ and ‘social interaction’ were explored. Reports of each code were saved in Microsoft Word and explored in their entirety and interpreted inductively. (4) Summaries were created of phase 3 code reports and grouped to produce final themes [54]. Themes were discussed and finalised with co‐authors, data in this paper are presented around these final three themes.

## 3 Findings

Participants described a range of symptoms with varying severities, as is typical with long Covid [14]. Many described disruption to their daily lives, including regarding employment and family life, with some so severely affected that they were unable to undertake ordinary daily tasks.

‘I'm still not able to work or do anything physical at all. I'm stuck at home at the moment and that's how it affects me, I can't do anything physical. I can't take my son to school, I can't go to work, I can't do any physical activities.’

Late 30s, Black Latino, Male

‘I didn't have the energy to do, you know, prepare a meal, or I couldn't stand up to brush my teeth or stand up in the shower, even that's why I was barely getting a shower at all. Just all sorts of really basic bits of self‐care’

Age NA, Black Asian, Female

The impact of intersectional dimensions on the lived experience of ethnic minority long Covid and their management of the condition is presented below. To present this intracategorical analysis, three major themes are explored in detail: *Gender; Socio‐economic factors; and Comorbidities and disabilities*. Whilst other intersectional issues are undoubtedly important for long Covid (e.g., age, religion and sexuality), these were not as prevalent among our participant narratives. Thus, they are not discussed in our paper (also see ‘Limitations’ section for discussion on this point). Whilst findings have been presented sequentially, in reality, dimensions will interact simultaneously, and in this section, we have highlighted examples where this occurs. All themes are supported by illustrative quote extracts.

### 3.1 Gender

Both men and women from ethnic minorities living with long Covid reported challenges in accessing support from their families and/or their wider communities, but for different reasons. For example, women in some ethnic minority communities felt they were deemed less important than men; thus, their health and well‐being were less of a priority. Alternatively, a lack of female representation in certain places (e.g., religious) could also make it difficult to gain support.

‘The negative perception about health plus women's health. If anything happened to men, they would say, “Oh yes, bless, oh poor…” they would have all the sympathy and everything.… Like, my mother‐in‐law … she doesn't have that concept that her daughter‐in‐law can get unwell.’

Early 50s, South Asian, Female

‘Interviewer: have you been able to speak to anyone at [place of worship]?

P: We don't have any female representatives. So, I don't.’

Early 50s, South Asian, Female

Regarding male narratives, barriers to accessing community support were more focused on preconceptions that seeking support was a sign of weakness and a source of shame and that participants needed to ‘man up’. This could promote a reluctance to seek support or discuss challenges they were facing, like financial concerns:

‘Every other person have whatever problem that they are having at that point in time, even the church, the church have programs. Everybody the government they have programs at times. So I feel like I don't wanna go there, anybody remind me, I think I should man up and take care of my myself.’ Early 30s, Mixed; Afro‐Caribbean, Male

‘I know people through support groups; that's about as far as it goes. I understand their situation, and I've got to admit, I've not been that open with what's happened to myself…. But the food bank—I've never, I've not told anyone about that at work, don't intend to.’

Early 60s, White‐Irish, Male

Gender could also interact with ethnicity to influence the support people reported receiving from partners to cope with their long Covid. While male narratives tended to focus on the positive support they received from their spouses, female participants focused on the lack of support they received from male partners. The latter could manifest as especially dismissive/questioning attitudes towards symptoms, lack of care of the woman when they were feeling unwell, or expectations that the woman's gendered duties (e.g., housework and childcare) would continue despite their illness. For some women, this could extend to insensitive remarks from their partner.

‘He's a lovely person, he's very loving, very empathetic. In terms of him, he hasn't always understood, I mean, he still doesn't always understand and there have even been times where he hasn't completely believed me as well. I think out of an effort to try and be helpful, you know, being like, “Are you sure that's what's going on? Are you sure it's not just you worrying about your symptoms?” and I get it, I do, but yes, he has done that a little bit, and yes, there have been times I've had to defend my choices about how I manage my fatigue.’

Early 30s, Middle Eastern, Female

‘Well, my wife has been everything for me, so she's been looking after my son and after me…. If it wasn't for me having my job and for my wife having to help me, then I don't know what would have happened to me.’

Late 30s, Black‐Latino, Male

In terms of encounters with health professionals, both male and female participants reported negative experiences. However, negative encounters and their impacts were more emphasised among women. Negative experiences included symptoms being dismissed as psychological in nature, delays in accessing care, long waits and not being taken seriously. Some experienced fears that their symptoms had worsened due to ensuing delays, others had found it hard to access sickness absence procedures due to a lack of medical endorsement:

‘I'd say not getting…. The right medical help early. You know if … if that pneumonia had been diagnosed at the beginning, I think that my lungs might be in a better place’

Mid‐50s, Asian, Female

Whilst men interpreted negative encounters with professionals as a result of being from an ethnic minority background, women tended to interpret them (additionally or solely) as being because of their gender.

‘I mean, being female, you don't get taken so seriously by doctors. They're very quick to label you as hysterical, and when I had pneumonia, they kept telling me I was just anxious and tried to put me on diazepam when I had pneumonia.’

Early 40s, British Indian, Female

In contrast to male narratives, female narratives tended to highlight a history of past numerous dismissive interactions with health professionals before long Covid. For example, one participant, when seeking care for long Covid, was dismayed to be referred to a consultant she had encountered pre‐long Covid and who had previously overlooked her medical needs. These repeated experiences of discrimination with healthcare providers before long Covid could mean that ethnic minority women are primed more than men to mistrust healthcare professionals.

‘They ended up referring me to [medical facility] but my concern was that I think one of the team, the consultant that my GP spoke to previously, because I wasn't seen as needing further treatment for this underlying health condition because of the mould and everything, I had lost trust in that particular consultant because he was part of that hospital team what I didn't want.’

Late 30s, British Indian, Female

### 3.2 Socio‐Economic Factors

Symptoms of long Covid adversely impact ability to work. Where participants were unable to work, accessing unemployment benefits within a system that lacks understanding of long Covid was reported to be challenging.

‘Financially, I'm impacted. My condition limits me in what I can do and what I can earn. It's devastating, it's destroyed my life.’

Late 30s, British Indian, Female

There were stark financial disparities reported within our ethnic minority sample. Some participants reported needing to return to work before they felt well enough for financial reasons (e.g., if they were on zero‐hours contracts), which could negatively impact recovery and contribute to anxiety. However, those in more supportive and flexible work environments could better manage some of the challenges of working with long Covid.

‘So, at that point, I was hopeless, you know, … I was coming behind on my bills…. So, I had to try to work some more or take more overtime, which stressed me out, it was a bad idea.’

Late 20s, Black Caribbean, Female

‘So, they're still there supportive and I'm still under review of occupational health nurse as well. And then every 2 weeks I had [a] meeting with my manager as well checking on me. So yeah touch wood I have been fortunate that I've been very lucky. I've been blessed.’

Mid 40s, Pakistani‐Asian, Male

Good financial resources allowed some participants to access specialised services that were difficult to get or unavailable in the NHS, including private medical healthcare, allied health professionals, psychological support, complementary therapy services and health supplements:

‘I asked to be seen by a neuropsychologist on the NHS and but they reject, they actually rejected my referral. And so I ended up paying for that privately, and so I was assessed by a neuropsychologist, and she's actually an expert in assessing patients with brain injury.’

Mid 30s, Mixed ethnicity, Male

‘Yes, acupuncture, I've tried something called safe and sound protocol. Safe and sound protocol, which is an auditory intervention, which is supposed to help the vagus nerve.’

Mid 30s, Bangladeshi, Male

Ethnic minority participants who had access to private healthcare routes were less focused on reporting poor experiences of healthcare due to perceived racism or sexism, and reported fewer experiences with delays and lack of communication using private services that were more characteristic of NHS settings:

‘No, no, I've never been discriminated, no. Healthcare, hospitals, hospitals is one of the places that you find out that love is given to everybody equally. Is about life and death…. It's very difficult to see maybe a medical practitioner that is a racist.’

30s, mixed Afro‐Caribbean, male

Better financial circumstances also gave some participants the opportunity to access practical help, providing some relief from everyday tasks:

‘I have a cleaner that comes twice a week now and I have also somebody who comes and cooks for me once a week, so basically, I'm able to throw money at various problems and just try and give myself a bit more time, and space’

Late 30s, British Indian, Female

In comparison, participants with fewer economic resources described how their lack of money was a considerable constraint to seeking further support, or how such assistance could lead to debt if they did seek it. Men in particular reported shame in having to access financial aid (e.g., food banks), while women focused on their general difficulties with finances, which at times could have a gendered dimension, as in the second quote below:

‘I've had to pay to get these things done, and it is shocking that I have to do that because that means, for those people who are not in a position, well, I'm not in a position financially to do it. I'm in debt because I'm desperate to get medical help.’

Late 30s, British Indian, Female

‘After COVID, because I didn't have a job and I had to get my prescription, my husband wouldn't give any money to me that is the reason I started doing [additional work], even I get so tired at the end of the day when I finish.… I get so tired, but I have to do it for the money’

Early 50s, South Asian, Female

However, working in the healthcare sector, which has a high percentage of ethnic minority employees, provided some participants with access to additional leverage in the health system and an insider understanding of how to get help for long Covid. For example, one participant talked about gaining early access to a long Covid clinic:

‘My manager referred me to the Long COVID clinics. So, in our workplace they had decided that there was a lot of people had been off with COVID and they had the long COVID symptoms. So because we are an [healthcare organisation] service and we've got, we've got access to nursing and all the kind of holistic care from psychologists, the physiotherapist, OTs, and they decided to trial a long COVID clinic for staff.’

Late 40s, British‐Pakistani, Male

### 3.3 Comorbidities and Disabilities

The presence of disabilities or other health conditions, in addition to long Covid, could further complicate the impact of long Covid on ethnic minority lives. Some participants reported pre‐existing health conditions which had been exacerbated by long Covid. Others reported developing new health conditions (e.g., overactive thyroid and pre‐diabetes) as an outcome of long Covid. Yet others believed their prior health condition left them more vulnerable to (and more impacted by) Covid‐19.

‘I'm suffering with fibromyalgia, aches and pains, and back…. I like cooking. Again, same thing I used to try, like, different cuisines, different dishes, different…. Now I just cook for the sake of it, like, obviously at the end of the day my daughter will be back from work and from school and husband from work, so I cook dinner. Like, if you would say you enjoy, no I don't much. I like walks which I enjoy, yes. But that limit, because of my pains and aches, I used to walk, like, hours but now I get so tired, and then yes, I can't do much, but I try to.’

Early 50s. South Asian, Female

‘Because of the [autoimmune disease] I'm very prone to picking up respiratory infections, which is why I picked up the COVID so quickly and I think that's why it affected me so hard.… I did have some limitations, but those have vastly increased since getting Covid.’

Early 40s, British Indian, Female

The presence of comorbidities, whatever their origin, added to symptom burden among ethnic minority participants. They also affected the impact of symptoms on daily living, ability to work, mental health and capacities to engage in activities to support recovery. Comorbidities could also mean needing more help and support, thus increasing the complexities and challenges of gaining support as discussed earlier, whether from health professionals, work or government benefits.

‘So, I saw a neuropsychiatrist. He's the one that diagnosed chronic fatigue, like, ME, stroke, yes, and he told me not to do anything. Okay. Just pace, he said, pace yourself…. And then I saw a rheumatologist about aches and pains, my back pain, foot pain, muscle pain…. And he said it's because I'm deconditioned. Yes, that's the word he used. It's because you're deconditioned, and you need to do lots of physical activities. The more physical, the better…. And I'm thinking, “What contradictory advice.” One tells me don't do anything and the other one's telling me do as much, do it in excess. Yes, so, that's why I said doctors are making me ill.’

Mid 50s, British Black Caribbean, Female

‘Your workplace are required to make adjustments for you if you're disabled.… So, I think I'm potentially in for more experience of ableism personally as, as I have to engage more with the, with the work and stuff.’

Mid 30s, Mixed ethnicity, Male

Past experiences of medical gaslighting regarding comorbidities were reported, in addition to those relating to medical help‐seeking for long Covid. This could lead to participants feeling as if they were in the wrong demographic to receive support. There could also be an increased illness burden related to associated treatment delays:

‘I was having lots of issues with it [menopause].… Nobody showed any sympathy. It's like it's a part of life just get on with it.… The support group [menopause] people are like, “I've been medically retired because of menopause.” Yes. Have a couple of hot sweats and there's all this help out here for me. Yes. I'm thinking okay, right, so a certain demographic has to just suffer through it, nobody cares, get on with it. And then another demographic, well here's all the help you can get.’

Mid 50s, British Black Caribbean, Female

## 4 Discussion

This qualitative secondary analysis explored the lived experiences of ethnic minorities along the lines of their various identities and social circumstances. Our results focused on an intracategorical interpretation of intersectionality (i.e., focusing on exploring lived experience and complexity therein). To do this, we focused on the experiences of ethnic minority participants with long Covid as a specific social group, and the role of other identities and positionings in turn. Our analysis confirmed that within our long Covid ethnic minority group of participants, the experience of living with the condition is shaped in profound ways along dimensions like socio‐economic status, presence of comorbidities and gender. In particular, individuals' financial capacity somewhat determined the type and extent of accessible care and support. Support levels from employers also influenced participants' ability to take adequate time to recover or work flexibility to accommodate long Covid symptoms. Thus, we suggest that there are both challenges and opportunities for ethnic minority agency in the inevitable complex mix of identities and positionality. The interplay of comorbidities along with long Covid also influenced the lived experience in complex ways, including heightening the risk of severe symptoms and complicating the overall management of long Covid. While both genders experienced challenges in gaining support from family and friends as well as health professionals, our data indicated challenges were gendered to some degree (e.g., men finding it hard to talk about their struggles, women feeling deprioritised due to their gendered position in the family, community and healthcare), with challenges being emphasised more in women's narratives. The findings of this study have thus confirmed the importance of considering the intracategorical intersectional dimensions of the lived experience of long Covid to reveal the deeper nuances in individual experiences of long Covid, as well as possibilities for agency.

The management of chronic conditions is substantially influenced by an individual's financial resources, particularly given the prolonged nature and the debilitating effects of conditions like long Covid [55]. Another key finding of our study was the importance of the intersection between ethnicity and socio‐economic status/work role, particularly in terms of the ability to access specialised secondary care, as well as more holistic treatments. Better financial resources enabled participants to explore medical and psychological support, as well as a range of allied and complementary therapies. Given the novel and chronic nature of long Covid, coupled with no definitive cure, exploration of alternative therapies may offer a way to manage specific symptoms [56, 57, 58]. The intersection of ethnic minority status, gender and socio‐economic status has been identified as a key factor in exacerbating health inequalities, particularly for women [59]. A recent report analysing the intersectional factors affecting women's living standards in the United Kingdom suggests that ethnic minority women are disproportionately affected by financial precarity, which can have negative implications for their overall health and well‐being [60]. Economic barriers can restrict agency in health‐related decision‐making, thereby exacerbating mental distress [61]. The findings of our study indicate that ethnic minority women with low financial autonomy and difficult relationships with their spouses are especially at risk when it comes to asserting control over healthcare decisions.

As highlighted in our study, the relationship dynamics between an employer and employee can determine the extent to which people are able to manage the challenges posed by long Covid that impact work‐related duties [62]. Here, adverse working conditions can contribute to a decline in health and well‐being [63]. Supportive workplace rehabilitation programmes provided by employers can positively facilitate the return to work for people recovering from long‐term illness [64, 65]. On the other hand, a hostile work environment, including stigmatisation of long‐term health conditions and unrealistic workload expectations, can negatively impact the ability of individuals to manage chronic illnesses [66]. In our study, participants with stable employment and supportive employers were able to balance work and recovery by utilising sick leave and flexible working arrangements. Moreover, direct support by the line manager can contribute to better management of chronic illness and enhance the overall well‐being of employees [67], as well as facilitate a smoother return‐to‐work transition and effective symptom management [68]. We found that individuals working in healthcare may specifically benefit from employer support, including better access to specialist services relevant to long Covid.

Our findings align with previous studies indicating that the presence of multiple comorbidities alongside long Covid significantly worsens mental well‐being, daily functioning and overall quality of life [69, 70, 71]. People with multiple chronic conditions often require care from multiple healthcare providers, and the complexity of overlapping multiple conditions can be overlooked as healthcare professionals tend to focus on single conditions [72]. In our study, we heard how a combination of health conditions/disabilities with conflicting treatment approaches had led to uncertainty about how to best manage health. The overlap of medical conditions can also exacerbate diagnostic delays, a well‐documented challenge for people from ethnic minorities [73]. Additionally, our participants reported experiencing ‘medical gaslighting’ related to their comorbidities and, they assumed, their socio‐demographic profile (especially their ethnicity). In the future, a critical disabilities perspective could be brought to bear on comorbidities and long Covid to shed light on how chronic health is not only experienced through the body, but is also very much shaped by vested power interests, ethnicities, institutional racism and other biases [74].

Previous studies have revealed unfair treatment of ethnic minority women during their healthcare interactions for various health conditions [75, 76, 77]. People from an ethnic minority, despite their gender, frequently encounter discrimination within healthcare settings [78], although the dismissal faced by ethnic minority women is often portrayed as their symptoms not being taken seriously [79]. As a newly emerging condition with limited understanding, long Covid increases individuals' vulnerability to dismissal of their symptoms regardless of their social identity and background [80]. However, our study suggests an additional risk of invalidation associated with being an ethnic minority and female [71, 81]. Less emphasis on negative health encounters by men in our study could be a result of less help‐seeking among male patients in general [82]. Difficult healthcare encounters can delay appropriate management support, which may negatively influence health outcomes. An investigation into the factors influencing the prognosis of a long‐term condition with similarities to long Covid ‐ myalgic encephalomyelitis/chronic fatigue syndrome ‐ revealed that delay in diagnosis can significantly worsen disease progression [83]. Moreover, the experience of dismissal can lead to ongoing mistrust towards healthcare professionals and discourage health‐seeking [84].

Emotional and practical support received from family and spouses can be crucial in managing chronic condition symptoms [85]. We found that some female participants perceived their male partners as less understanding and, at times, outright unsupportive. On the other hand, male narratives described the spousal support they received as essential in coping with long Covid. Additionally, ethnic minority women described less support from families and communities compared to their male counterparts, with women feeling undervalued, misunderstood and that their well‐being was not a priority. Similarly, a study examining family support among African American women with type 2 diabetes found that women often felt their needs and challenges were not fully understood by their social networks, which diminished the effectiveness of the support they received [86]. Lack of social support can also increase the stigma associated with chronic unexplained conditions [87]. The presence of stigma associated with an illness is commonly reported among ethnic minority women and may contribute to the underutilisation of available services [88]. In the case of male participants, we found some perceptions of seeking care as a weakness and thus shameful, which was consistent with traditional notions of masculinity. The strong internalisation of societal norms of traditional forms of masculinity among Black men has been shown to hinder their willingness to seek timely care for chronic conditions [89]. Our findings are consistent with other research that suggests masculine norms, when combined with the experience of racial discrimination, may further discourage individuals from seeking help [90].

Long Covid is an emerging illness, and NICE guidance [4] was based on early studies, which were limited by a lack of inclusion of people from minority ethnic groups [91]. Revised guidelines and the delivery of safe and equitable person‐centred care need to be responsive to the lived experiences of people in all ethnic minority groups. Strategies to tackle ethnic inequalities in healthcare require an evaluation and reduction of the individual, systemic and structural obstacles to good care. Additionally, improving health services to support patients with conditions that have limited treatment options and health professional care is vital. Social prescribers are a group of health professionals who can potentially reach out to disadvantaged communities [91], and our ongoing research programme explores how this group of professionals can be better supported to work with underserved populations living with long Covid. However, whilst education and training of individual clinicians are important to prevent ‘medical gaslighting’ within the consultation, the availability of services for people with long Covid is also vital. The opposite is playing out with services for people with long Covid in the United Kingdom, which are being decommissioned [92], with the likelihood of causing further disparities in care.

## 5 Limitations

The original purpose of the data collection for this project was exploratory, to understand the broad experiences of ethnic minority people living with long Covid. The intersectionality perspective was not specifically incorporated into the data collection plan or tool, and therefore, data may not cover all the intersectional experiences participants faced. Additionally, with limited resources for our study, we were not able to actively recruit participants from diverse genders (about 0.5% of men and women identify as non‐binary or trans overall [93]) and sexualities; therefore, we have not been able to explore these important experiences. Little is known about the experiences of long Covid among those from sexual and gender diverse people. Given such communities often experience barriers to healthcare, for example via assumptions of heteronormativity, experiences of minority stress (i.e., chronic stress due to ongoing prejudiced treatment), or experiences of victimisation and discrimination [94], their perspectives would be an important topic for future research. It is also important to acknowledge that ethnic minority groups are not monolithic; instead, they are diverse internally. Whilst initially the original study aimed to focus on specific ethnicities, the PPI representatives advised us to include broad experiences due to the limited research on long Covid and ethnic minorities. Thus, we recruited widely. However, we have ensured that we have not treated participants as singular by including the self‐described ethnicities for each participant quoted. Subsequent research should explore long Covid experiences in detail within groups, such as ‘South Asian’. This approach may create greater depth and further insights into this topic. However, we note that even focusing on ‘South Asian’ participants will cover many different ethnic groups and provide immensely rich and complex data. Additionally, it should be noted that the intracategorical approach to intersectionality can be critiqued as resembling descriptive narrative. The authors acknowledge that the approach can create challenges for presenting deeper, nuanced intersectional experiences that consider theories about structural power dynamics as well as histories shaping those intersections. However, we argue that our approach of considering multiple categories provides missing perspectives that are neglected by focusing on singular categories. It also humanises intersectionality for wider audiences, while challenging assumptions about singular identity categories themselves. Due to the current paper being a secondary analysis, the advisory board had disbanded before the current paper was written, and there was no additional funding, including for their involvement. Thus, this paper did not benefit from their feedback, although earlier phases of the research did (e.g., recruitment, interview guide and consideration of emerging themes).

## 6 Conclusions

This study makes a significant contribution to understanding long Covid amongst ethnic minorities by examining the complex and often overlooked intersectional experiences of ethnic minority individuals, who are frequently marginalised or treated as a unified group in research. Despite its limitations, this study has shed light on the fact that experiences of living with long Covid may vary significantly along various dimensions within ethnic minority communities. Key intersections of importance include gender, socio‐economic status and comorbidities. Findings indicate that more needs to be done to promote equality in healthcare, including revised care guidelines that incorporate ethnic minority experiences, improved strategies to tackle obstacles to care, and increased availability of long Covid‐specific services.

## Author Contributions

**Yojana Lotankar:** formal analysis, writing – original draft, writing – review and editing. **Anna Cheshire:** formal analysis, supervision, writing – original draft, writing – review and editing, visualization. **Damien Ridge:** conceptualization, investigation, funding acquisition, writing – original draft, methodology, visualization, writing – review and editing, formal analysis, supervision. **Carolyn Chew–Graham:** writing – review and editing, conceptualization, funding acquisition. **Nina Symth:** conceptualization, investigation, funding acquisition, writing – original draft, methodology, visualization, writing – review and editing, formal analysis, supervision. **Claudia R Knowles:** writing – review and editing. **Dipesh Gopal:** writing – review and editing, funding acquisition. **Tom Kingstone:** funding acquisition, writing – review and editing. **Shoba Dawson:** writing – review and editing.

## Disclosure

The views expressed are those of the authors and not necessarily those of NIHR or the Department of Health and Social Care.

## Conflicts of Interest

The authors declare no conflicts of interest.

## Supporting information

Supplementary Information

## Data Availability

The data are not publicly available due to privacy or ethical restrictions.
