# Supplementary material for: Intersectionality and Long Covid: Understanding the Lived Experiences of Ethnic Minority Groups in the United Kingdom
Source: Health Expect. 2025 Sep 4;28(5):e70413. doi: 10.1111/hex.70413 (PMC12409300; doi:10.1111/hex.70413)
Supplement: Supplementary file 1 — Supplementary Information [file HEX-28-e70413-s001.docx]

**Positionality statements – authors and interviewers**

**Authors and interviewers**

DR is a first-generation university graduate and migrant from Australia, who is also part of the LGBTQ+ community, and has a keen interest in marginalised communities and their experiences of healthcare and discrimination and has most recently published on medical ambivalence facing ethnic minority patients, as well as LGBTQ+ people and antibiotic resistance.

NS is female and has a keen interest in research and strategies to enhance people’s health and wellbeing. She has worked in healthcare previously and has close personal relationships with people who work in healthcare, providing insights into the workings of healthcare and impacts on people who work within it and use the service. She acknowledges her ability to navigate the healthcare system in her position as a White, educated, middle class position and is passionate to make healthcare equitable for all from different backgrounds.

CK is White-female, educated to postgraduate level, who has not had an experience of long-COVID. As ex-NHS administrator who endeavours to return to the NHS as a trained Counselling Psychologist, she acknowledges her feminist views and personal interest in the sociological and political goal of reducing disparities in healthcare.

SD is a first generation, South Asian, female, researcher whose work focuses on research inclusion and addressing health inequities. She brings her personal and professional perspectives to the interpretation of data.

**Authors**

As a South Asian, female and qualitative researcher with an interdisciplinary background in medical anthropology and public health, YL brings both her personal and professional perspectives to this analysis. She conducted the secondary analysis of the anonymised transcripts provided by the research team and was not involved in the original data collection. Her lived experience as a minority ethnic person informs her sensitivity to the issues discussed by participants.

AC is a senior researcher with a background in health psychology, who is committed to research that seeks to understand and improve the lives of those living with long-term conditions. Having lived with a condition similar to long-COVID for 30 years, she is passionate about raising the profile and understanding of the long-COVID, as well as improving healthcare experiences of all with the condition, from her position as a White, educated, middle-class female.

CCG is a general practitioner and researcher. She is White and would describe herself as middle-class, although brough up in working class family as a child. She has conducted a lot of research on persistent physical symptoms, such as CFS/ME, irritable bowel syndrome, chronic pelvic pain. She hopes that her research will lead to improved patient / service user experiences and reduced health disparities.

DG is South Asian male researcher who works a general practitioner (GP) and primary care researcher. He is informed by his personal experience of coming from an ethnic minority background and professional experience of treating people with Long COVID and those from an ethnic minority background. His work focuses on tackling health inequalities and lives of people with a cancer diagnosis.

TK is a White British male educated to PhD level with a background in psychology, sociology and critical gerontology. He has worked in research for 15 years in a variety of multidisciplinary teams alongside clinical, academic and public partners. Through his research he has sought to explore lived experiences of long-term conditions (including common mental health problems, long covid), uncover barriers to help-seeking (including stigma, health disparities), and apply a critical lens on healthcare service design and delivery.

**Interviewers only**

JG is female form south Asian background, completing postgraduate training in health psychology with a keen interest in qualitative methods and equitable healthcare access for marginalised groups.
